# Sevelamer crystals—an unusual cause of large bowel obstruction

**DOI:** 10.1093/jscr/rjab228

**Published:** 2021-06-16

**Authors:** Hannah C Cockrell, Sarah Cottrell-Cumber, Kathryn Brown, Jason G Murphy

**Affiliations:** Department of Surgery, University of Mississippi Medical Center, Jackson, MS, USA; Department of Surgery, University of Mississippi Medical Center, Jackson, MS, USA; Department of Pathology, Mississippi Baptist Hospital, Jackson, MS, USA; Surgical Clinic Associates, Mississippi Baptist Hospital, Jackson, MS, USA

## Abstract

Sevelamer is a common phosphate binder used to manage hyperphosphatemia in end-stage renal disease. The medication has a well-documented gastrointestinal side-effect profile including nausea, vomiting and abdominal pain. There are few case reports of Sevelamer crystal deposition causing gastrointestinal mucosal injury, pseudotumor or obstruction. Here, we discuss a patient on Sevelamer who required operative management of a sigmoid obstruction. Surgical pathology showed pericolonic abscess with Sevelamer crystals.

## INTRODUCTION

Management of hyperphosphatemia in patients with end-stage renal disease reduces the risk of vascular calcification, endothelial damage and mortality [1]. Dietary modification and oral phosphate binders are the mainstay of treatment [1]. Sevelamer is a non-absorbable ion-exchange resin that binds phosphate in the gastrointestinal tract [2]. Known side-effects include nausea, vomiting and abdominal pain [3, 4]. Rarely, Sevelamer crystals deposit in the gastrointestinal tract causing mucosal ulceration or colonic obstruction.

## CASE REPORT

**Figure 1:**
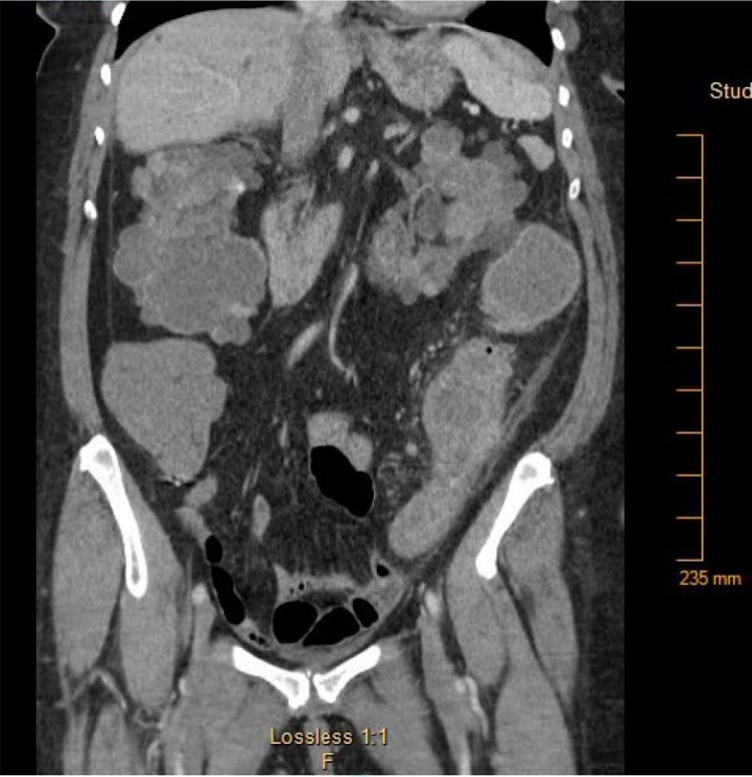
Computed tomography of the abdomen and pelvis showing focal narrowing of the sigmoid colon with proximal colonic dilatation.

**Figure 2:**
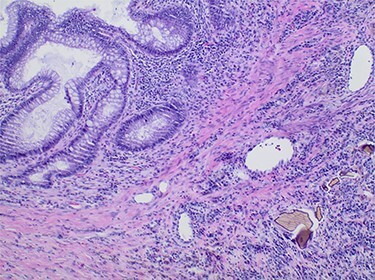
Pathology slide of the sigmoid colon with Sevelamer crystals; colonic mucosa (upper left) with adjacent inflammatory reaction containing entrapped sevelamer crystals (lower right).

A 65-year-old African American female with history of end-stage renal disease secondary to autosomal dominant polycystic kidney disease presented our Emergency Department with abdominal pain and hematochezia. She was evaluated with esophagogastroduodenoscopy and colonoscopy, which showed multiple non-bleeding sigmoid diverticula and descending colon mucosal erosions consistent with colitis. She was treated medically with metronidazole and ciprofloxacin. Her symptoms recurred 1-week after hospital discharge. She complained of intermittent, severe left lower quadrant pain with associated nausea and non-bilious emesis. Computed tomography (CT) on re-admission showed thickening of the descending-sigmoid colon junction with fluid-filled, dilated colon proximally (Fig. 1). Given symptoms and CT imaging consistent with early large bowel obstruction, the patient was taken to the operating room for exploratory laparotomy, sigmoid colectomy and end colostomy. Surgical pathology revealed pericolonic abscess and Sevelamer crystals (Fig. 2). The patient’s Sevelamer was discontinued, and her postoperative course was uncomplicated. She was seen back for planned elective colostomy reversal 4 months after her initial operation.

## DISCUSSION

This case adds to the limited literature on Sevelamer-induced gastrointestinal mucosal injury and obstruction. The first histologic description of Sevelamer crystals in the gastrointestinal tract was published by Swanson *et al.* They identified non-polarizable ‘fish-scale’ crystals with a yellow–red appearance on hematoxylin and eosin staining. Crystal deposits were associated with mucosal injury, ulceration and inflammatory polyps. The authors suggested a positive correlation between Sevelamer dosing and extent of mucosal injury but were unable to establish direct causation given their small case series [2].

Subsequent case reports have supported a causal relationship between Sevelamer use and gastrointestinal mucosal ulceration. Chintamaneni *et al.*, e.g. described a 61-year-old female patient with end-stage renal disease on Sevelamer for hyperphosphatemia who was admitted with painless hematochezia. She was found to have a sigmoid ulceration on colonoscopy, and biopsy of the ulcer revealed Sevelamer crystals [3]. Similarly, Nambiar *et al.* reported a patient with Sevelamer-related hepatic flexure ulceration [4].

There are fewer references in the literature about Sevelamer concretion resulting in colonic pseudotumor or large bowel obstruction. One published case report describes a 79-year-old male who was admitted with abdominal pain and diarrhea and was found to have CT imaging with circumferential cecal thickening concerning for a colonic neoplasm. On colonoscopy, however, the patient was discovered to have a partially obstructing polypoid mass. Pathologic examination showed Sevelamer crystals with no background dysplasia or adenocarcinoma [5].

While rare, gastrointestinal complications are seen in end-stage renal disease patients with long-term Sevelamer use. Sevelamer crystal deposition should be considered as an underlying pathology in those patients with kidney disease who present with painless hematochezia or symptoms of large bowel obstruction.

## CONFLICT OF INTEREST STATEMENT

None declared.

## FUNDING

None.
